# Supplementary material for: CAVE: Connectome Annotation Versioning Engine
Source: Nat Methods. 2025 Apr 9;22(5):1112–20. doi: 10.1038/s41592-024-02426-z (PMC12074985; doi:10.1038/s41592-024-02426-z)
Supplement: Supplementary file 1 — Supplementary Tables 1 and 2. [file 41592_2024_2426_MOESM1_ESM.pdf]

nature methods

Supplementary information https://doi.org/10.1038/s41592-024-02426-z

CAVE: Connectome Annotation Versioning Engine

In the format provided by the authors and unedited

Package	Description	github
CAVEdeployment	Kubernetes deployment scripts	github.com/CAVEconnectome/CAVEdeployment
ChunkedGraph	Proofreading & Meshing service	github.com/CAVEconnectome/PyChunkedGraph
AnnotationEngine	Annotation service	github.com/CAVEconnectome/AnnotationEngine
AnnotationDB	Annotation storage	github.com/CAVEconnectome/DynamicAnnotationDB
Materialization	Materialization service	github.com/CAVEconnectome/MaterializationEngine
EMAnnotationSchemas	Annotation schemas	github.com/CAVEconnectome/EMAnnotationSchemas
MiddleAuth	Auth service	github.com/CAVEconnectome/middle_auth
MittleAuth Client	Client interface for the auth service	github.com/CAVEconnectome/middle_auth_client
Info	Info service	github.com/CAVEconnectome/AnnotationFrameworkInfoService
StateServer	Neuroglancer state storage	github.com/CAVEconnectome/NeuroglancerJsonServer
L2Cache	Chunkedgraph cache	github.com/CAVEconnectome/PCGL2Cache
Guidebook	Proofreading guidance	github.com/CAVEconnectome/Guidebook
DashOnFlask	Dash app deployment	github.com/CAVEconnectome/dash_on_flask
CAVEcanary	Error detection and notification system	github.com/CAVEconnectome/CAVEcanary
datastoreflex	Hybrid Datastore and GCS interface	github.com/CAVEconnectome/datastore-flex
MeshParty	Mesh python client	github.com/CAVEconnectome/MeshParty
cloudvolume	Image and mesh interface	github.com/seung-lab/cloud-volume
cloudfiles	Storage interface	github.com/seung-lab/cloud-files
pcg_skel	ChunkedGraph-based skeletonization	github.com/CAVEconnectome/pcg_skel
NGLAnnotationUI	Create ngl states programmatically	github.com/CAVEconnectome/nglui
neuroglancer	Interactive UI for image and mesh data	github.com/seung-lab/neuroglancer<br>github.com/google/neuroglancer
ngextend	Ngl wrapper to add customizations	github.com/seung-lab/ng-extend

Supplementary Table 1: Overview of CAVE-related packages. Most packages are managed in the following GitHub team: https://github.com/CAVEconnectome.

ID	First Author	Year	X (µm)	Y (µm)	Z (µm)	Vol. (mm3)	Species	Reconstruction	Est.	Proof-Tool	Sup.
1	Bock	2011	450	330	50	0.00743	Mouse	manual			
2	Brigmann	2011	350	350	60	0.00735	Mouse	manual			
3	Helmsteader	2013	132	114	80	0.00120	Mouse	manual			
4	Takemura	2013	37	37	71	0.00010	Drosophila	semi-automated	x	Raveler	
5	Kim	2014	350	350	60	0.00735	Mouse	semi-automated		Eyewire	1
6	Kasthuri	2015	40	40	50	0.00008	Mouse	semi-automated		Mojo/Dojo	
7	Takemura	2015	21.4	29.6	39.9	0.00003	Drosophila	semi-automated	x	Neutu	
8	Morgan	2016	400	600	280	0.06720	Mouse	manual			
9	Lee	2016	450	450	150	0.03038	Mouse	manual			
10	Wanner	2016	118	108	72	0.00092	Zebrafish	manual			
11	Ohyama	2016	80	60	50	0.00024	Drosophila	manual			
12	Brigmann	2016	260	210	50	0.00273	Mouse	manual			
13	Kornfeld	2017	166	166	77	0.00212	Zebrafinch	manual			
14	Schmidt	2017	424	429	274	0.04984	Rat	manual			
15	Takemura	2017	40	50	120	0.00024	Drosophila	semi-automated		Neutu	
16	Takemura	2017	40	40	80	0.00013	Drosophila	semi-automated		Neutu	
17	Zheng	2018	750	350	250	0.02780	Drosophila	manual	x		
18	Svara	2018	207	74	74	0.00113	Zebrafish	manual			
19	Bloss	2018	200	300	17	0.00102	Mouse	manual			
20	Drawitsch	2018	130	110	85	0.00122	Mouse	manual			
21	Motta	2019	62	95	93	0.00055	Mouse	manual			
22	Microns	2019	250	140	90	0.00315	Mouse	semi-automated		CAVE	
23	Wilson	2019	190	120	49.74	0.00113	Mouse	manual			
24	Wilson	2019	190	120	75.42	0.00172	Mouse	semi-automated		Mojo/Dojo	
25	Karimi	2020	96	134	68	0.00087	Mouse	manual			
26	Karimi	2020	87	152	68	0.00090	Mouse	manual			
27	Karimi	2020	56	213	88	0.00105	Mouse	manual			
28	Karimi	2020	142	91	71	0.00092	Mouse	manual			
29	Kornfeld	2020	114	98	96	0.00107	Zebrafinch	semi-automated		Knossos	
30	Dorkenwald	2020	750	350	250	0.02780	Drosophila	semi-automated	x	CAVE	17
31	Scheffer	2020	250	250	250	0.01563	Drosophila	semi-automated		Neutu	
32	Phelps	2021	950	350	200	0.01640	Drosophila	manual	x		
33	Shapson-Coe	2021	3000	2000	150	0.44296	Human	semi-automated	x	CAVE	

34	Microns	2021	1400	870	800	0.97440	Mouse	semi-automated		CAVE	
35	Gour	2021	127	104	81	0.00107	Mouse	manual			
36	Loomba	2022	164	105	108	0.00186	Mouse	manual			
37	Loomba	2022	167	218	106	0.00386	Macaque	manual			
38	Loomba	2022	167	219	106	0.00388	Macaque	manual			
39	Loomba	2022	166	216	112	0.00402	Human	manual			
40	Loomba	2022	170	215	79	0.00289	Human	manual			
41	Svara	2022	700	450	320	0.05040	Zebrafish	semi-automated	x	Knossos	
42	Vishwanathan	2022	250	120	80	0.00240	Zebrafish	semi-automated		Eyewire	
43	Azevedo	2022	950	350	200	0.01640	Drosophila	semi-automated	x	CAVE	32
44	Takemura	2023	950	350	200	0.01400	Drosophila	semi-automated	x	Neutu	
45	Bidel	2023	390	260	27	0.00274	Octopus	manual			

Supplementary Table 2. Released connectomics datasets, their sizes and reconstruction methods. We estimated sizes for a few datasets based on figures or available volume meshes. In those cases, the dimensions may describe a larger volume than the actual imaged and reconstructed volume. These are indicated in the “Est.” column. A few datasets were published with different reconstructions. These relationships are indicated in the supersedes (“Sup.”) column.
